# Prenatal and postnatal mothering by diesel exhaust PM_2.5_-exposed dams differentially program mouse energy metabolism

**DOI:** 10.1186/s12989-017-0183-7

**Published:** 2017-01-18

**Authors:** Minjie Chen, Shuai Liang, Huifen Zhou, Yanyi Xu, Xiaobo Qin, Ziying Hu, Xiaoke Wang, Lianglin Qiu, Wanjun Wang, Yuhao Zhang, Zhekang Ying

**Affiliations:** 1 Department of Environmental Health, School of Public Health, Fudan University, Shanghai, 200032 China; 2 Department of Medicine Cardiology Division, School of Medicine, University of Maryland, 20 Penn St. HSFII S022, Baltimore, MD 21201 USA; 3 Department of Bile Pancreatic Surgery, Xiangya Hospital, Central South University, Changsha, Hunan 410008 China; 4 Department of Pathology, Hubei University of Science and Technology, Xianning, Hubei 437100 China; 5 Department of Endocrinology, the People’s Hospital of Zhengzhou University (Henan Provincial People’s Hospital), Zhengzhou, Henan 450003 China; 6 Department of Occupational and Environmental Health, School of Public Health, Nantong University, Nantong, 226019 China; 7 Department of Neurology, Zhongshan Hospital, Fudan University, Shanghai, 200032 China

**Keywords:** Diesel exhaust PM_2.5_, Maternal exposure, Obesity, Developmental programming

## Abstract

**Background:**

Obesity is one of the leading threats to global public health. It is consequent to abnormal energy metabolism. Currently, it has been well established that maternal exposure to environmental stressors that cause inappropriate fetal development may have long-term adverse effects on offspring energy metabolism in an exposure timing-dependent manner, known as developmental programming of health and diseases paradigm. Rapidly increasing evidence has indicated that maternal exposure to ambient fine particles (PM_2.5_) correlates to abnormal fetal development. In the present study, we therefore assessed whether maternal exposure to diesel exhaust PM_2.5_ (DEP), the major component of ambient PM_2.5_ in urban areas, programs offspring energy metabolism, and further examined how the timing of exposure impacts this programming.

**Results:**

The growth trajectory of offspring shows that although prenatal maternal exposure to DEP did not impact the birth weight of offspring, it significantly decreased offspring body weight from postnatal week 2 until the end of observation. This weight loss effect of prenatal maternal exposure to DEP coincided with decreased food intake but not alteration in brown adipose tissue (BAT) morphology. The hypophagic effect of prenatal maternal exposure to DEP was in concord with decreased hypothalamic expression of an orexigenic peptide NPY, suggesting that the prenatal maternal exposure to DEP impacts offspring energy balance primarily through programming of food intake. Paradoxically, the reduced body weight resulted from prenatal maternal exposure to DEP was accompanied by increased mass of epididymal adipose tissue, which was due to hyperplasia as morphological analysis did not observe any hypertrophy. In direct contrast, the postnatal mothering by DEP-exposed dams increased offspring body weight during lactation and adulthood, paralleled by markedly increased fat accumulation and decreased UCP1 expression in BAT but not alteration in food intake. The weight gain induced by postnatal mothering by DEP-exposed dams was also expressed as an increased adiposity. But it concurred with a marked hypertrophy of adipocytes.

**Conclusion:**

Prenatal and postnatal mothering by DEP-exposed dams differentially program offspring energy metabolism, underscoring consideration of the exposure timing when examining the adverse effects of maternal exposure to ambient PM_2.5_.

## Background

Obesity is one of the leading threats to global public health [1]. Numerous studies have demonstrated that it may originate from early-life exposure to environmental stressors that cause inappropriate fetal and/or neonatal development, referred to as the developmental programming of health and diseases (DOHaD) paradigm [2]. Air pollution is one of the leading preventable threats to global health [3]. A rapidly increasing number of epidemiological studies have shown that prenatal exposure to ambient fine particulate matter with a diameter ≤ 2.5 μm (PM_2.5_) is associated with a variety of manifestations of abnormal fetal development such as abortion, placental dysfunction, low birth weight and pre-term birth [4–12]. Toxicological studies have also demonstrated that gestational exposure to concentrated ambient PM_2.5_ or diesel exhaust impacts fetal and/or placental development in a variety of animal models [13–19]. According to the DOHaD paradigm, these demonstrations of disruption of fetal development by gestational exposure to PM_2.5_ strongly suggest that it may be a risk factor for developmental programming of diseases encompassing obesity. Supporting this, gestational exposure to ambient pollutants have been shown to increase body weight [13, 14], and aggravate high fat diet-induced obesity [20]. However, how gestational PM_2.5_ exposure impacts energy metabolism and subsequently adiposity in adult offspring has not yet systemically investigated.

In addition to the gestation period, infancy has been shown to be vulnerable to developmental programming by environmental stressors [2]. Furthermore, several studies have indicated that the timing of exposure to environmental stressor determines not only the severity but also the nature of developmental programming [2]. For example, maternal exposure to famine in early gestation results in increased body mass index (BMI), whereas exposure to famine in late gestation and early infancy leads to decreased BMI [21]. Interestingly, while several studies have demonstrated adverse health effects of gestational exposure to PM_2.5_, as mentioned above, few studies have investigated whether postnatal mothering by PM_2.5_-exposed dams programs offspring diseases.

Given that both PM_2.5_ pollution and obesity will continue to be the leading health concerns in the foreseeable future, additional studies are warranted to document the developmental programming of energy metabolism by maternal exposure to PM_2.5_ and its timing-dependency. In the present study, we therefore examined the long-term effects of prenatal and postnatal mothering by diesel exhaust PM_2.5_ (DEP)-exposed dams on offspring development and energy metabolism. Our results unexpectedly showed different developmental programming of energy metabolism by prenatal and postnatal mothering of DEP-exposed dams, and thus not only raised more health concerns about maternal exposure to PM_2.5_ but also underscored consideration of the timing of exposure when examining the health effects of maternal exposure to air pollutants.

## Methods

### Animals

University of Maryland, Baltimore (UMB) is an AAALAC accredited institution. All procedures of this study were approved by the Institutional Animal Care and Use Committee (IACUC) at UMB, and all the animals were treated humanely and with regard for alleviation of suffering. C57Bl/6j mice (4-week-old, 12 male and 12 female) were purchased from the Jackson Laboratories (Stock #000664) and were housed in animal facilities at UMB, which maintained the 12-h light/12-h dark light cycle and the temperature and humidity within the recommended limits. Breeding cages were set up with one male and one female at the age of 12 weeks. Offspring were weaned once they were 3 weeks old.

### Maternal DEP intratracheal instillation

DEP were obtained from the National Institute of Standards and Technology (DEP; SRM 2975; NIST, Gaithersburg, MD, USA). They were stored at 4 °C and kept away from direct sunlight. To perform instillation, they were suspended in sterile normal saline. Prior to removal of subsamples for analysis, the contents of the bottle were mixed thoroughly. To minimize aggregation, particle suspensions were sonicated (Clifton Ultrasonic Bath, Clifton, NJ, USA) for 20 min on the day of instillation and vortexed 30 s before each instillation. The dams were the only members of the pair instilled. The instillation of DEP was performed as previously described with minor modifications [22]. Briefly, to instill DEP, the animals were anesthetized with 3% isoflurane, and placed supine with extended neck on an angled board. A Becton Dickinson 18 Gauge cannula was inserted via the mouth into the trachea. DEP suspension (20 μg in 50 μl, representing an average daily dose of 8.6 μg/mouse and approximately equating to inhalational exposure to 160 μg/m^3^ PM_2.5_) [23] or saline (50 μl) were intratracheally instilled via a sterile syringe and followed by an air bolus of 150 μl. The intubation catheter was removed and the mouse transferred to a vertical hanging position with the head up for 5 min, ensuring that the delivered material was maintained in the lung and did not block the airways. The deposition and distribution of instilled material was verified by installing Evans blue (data not shown). Either DEP or saline was instilled 3 times/week (Monday, Wednesday, and Friday) beginning at the age of 5 weeks and ending once offspring were weaned. As mating started at the age of 12 weeks, there was approximately a 7-week pre-conceptional instillation.

### Analysis of offspring growth trajectory and food intake

The body weights of offspring were measured weekly from birth until 16 week old. Food intake assessment was performed when they were 18–20 week old. Briefly, each mouse was housed in one normal cage, and the weight of diet was recorded daily for 7 consecutive days. Food intake was calculated as the difference between two consecutive days. The average food intake of the last five days was presented. All offspring were euthanized when they were 20–22 weeks old.

### Histological analysis

Epididymal adipose tissue and brown adipose tissue (BAT) were fixed in 4% paraformaldehyde, embedded in paraffin, cut into 5-μm sections, and stained with hematoxylin and eosin. The histology sections were viewed at 20× magnification, and images were obtained with a SPOT digital camera (Diagnostic Instruments, Sterling Heights, MI) by one person who was blind to the grouping. The total number and cross-sectional areas of adipocytes in epididymal adipose tissues were calculated as previously described. The fat droplet areas of BAT were obtained using Imagej software, and the results were expressed as the percentage of total area.

### Real-time RT-PCR

Total RNA was isolated from tissues (epididymal adipose tissue and hypothalamus) with TRIzol reagent (Invitrogen, Carlsbad, CA, USA). 2 μg total RNA was reverse transcribed using random hexamers and the ThermoScript RT-PCR System (Invitrogen). Quantitative RT-PCR was performed with the Stratagene Mx3005 using SYBER Green PCR Master Mix (Applied Biosystems, Carlsbad, CA, USA). The sequences of primers were presented in Table 1. The relative expression level was obtained as described previously [24]. Briefly, Ct values were acquainted through analysis with software provided by the manufacturer, and differences of Ct value between target gene and GAPDH (∆Ct) and then 2^∆Ct^ were calculated.

**Table 1:** PCR Primers

Gene Name	Acronym	Function	Forward primer	Reverse primer
glyceraldehyde-3-phosphate dehydrogenase	GAPDH	a house-keeping gene encoding enzyme for the oxidative phosphorylation of glyceraldehyde-3-phosphate	TGAACGGGAAGCTCACTGG	TCCACCACCCTGTTGCTGTA
pro-opiomelanocortin-alpha	POMC	a polypeptide hormone precursor involved in energy homeostasis	GCCCTCCTGCTTCAGACCTC	CGTTGCCAGGAAACACGG
neuropeptide Y	NPY	a neuropeptide that regualtes food intake	TACCCCTCCAAGCCGGACAA	TTTCATTTCCCATCACCACATG
agouti related neuropeptide	AgRP	a neuropeptide that regulates feeding behavior	CGGAGGTGCTAGATCCACAGA	AGGACTCGTGCAGCCTTACAC
interleukine-1beta	IL-1b	a pro-inflammatory cytokine	ACGGACCCCAAAAGATGAAG	TTCTCCACAGCCACAATGAG
interleukine-6	IL-6	a pro-inflammatory cytokine	ATCCAGTTGCCTTCTTGGGACTGA	TAAGCCTCCGACTTGTGAAGTGGT
tumor necrosis factor alpha	TNFα	a pro-inflammatory cytokine	TTCCGAATTCACTGGAGCCTCGAA	TGCACCTCAGGGAAGAATCTGGAA
suppressor of cytokine signaling 3	SOCS3	cytokine-inducible negative regulators of cytokine signaling	GCGGGCACCTTTCTTATCC	TCCCCGACTGGGTCTTGAC
Leptin		an adipokine involved in regulation of body weight	GGGTAATACTTAAACAGTGACC	CTATCTGAAAATAAAAACTTCATG
Adiponectin		an adipokine involved in regulation of fatty acid catabolism and glucose levels	AGGGAGAGAAAGGAGATGCAG	CTTTCCTGCCAGGGGTTC
fatty acid synthase	FAS	the synthesis of palmitate from acetyl-CoA and malonyl-CoA	TGCTCCCAGCTGCAGGC	GCCCGGTAGCTCTGGGTGTA
peroxisome proliferator activated receptor gamma	PPARγ	a regulator of adipocyte differentiation.	TCGCTGATGCACTGCCTATG	GAGAGGTCCACAGAGCTGATT
sterol regulatory element binding transcription factor 1c	SREBP-1c	a transcription factor that binds to the sterol regulatory element-1 (SRE1), which is involved in sterol biosynthesis	GATGTGCGAACTGGACACAG	CATAGGGGGCGTCAAACAG
Acetyl-CoA carboxylase	ACC	A biotin-containing enzyme which catalyzes the rate-limiting step in fatty acid synthesis.	GCCGTGGGGAAGGAAAAGT	CTCCTGGTTGATGCTCGACA
PPARG coactivator 1 alpha	PGCα	a transcriptional coactivator involved in energy metabolism.	GAGAATGAGGCAAACTTGCTAGCG	TGCATGGTTCTGAGTGCTAAGACC
estrogen receptor 1 (alpha)	ER	a member of the nuclear hormone family of intracellular receptors	ACCATTGACAAGAACCGGAG	CCTGAAGCACCCATTTCATT
CCAAT/enhancer binding protein alpha	CEBP	a transcription factor involved in body weight homeostasis.	CTGCGGGGTTGTTGATGT	ATGCTCGAAACGGAAAAGGT
Preadipocyte factor 1	PREF1	Inhibits Adipogenesis	AGTGCGAAACCTGGGTGTC	GCCTCCTTGTTGAAAGTGGTCA

### Tissue harvesting, western blotting and leptin protein assessment

Animals were fasted overnight and i.p. injected with insulin (10 U/kg body weight). After 20 min, animals were euthanized by overdose of isoflurane. Blood was collected from heart and centrifuged at 3000 rpm for 5 min. Plasma was immediately stored in dry ice and then −80 °C. Hypothalamus was isolated as described before [25], and then snap-frozen in liquid nitrogen. All tissues were stored at −80 °C until further processing. Lysates of brown adipose tissue were prepared using RIPA buffer (Sigma, St. Louis, MO) supplemented with protease and phosphatase inhibitors (Sigma, St. Louis, MO). Protein samples were then separated by 10% SDS-polyacrylamide gel electrophoresis and electroblotted onto polyvinylidene fluoride membranes. Target protein was detected by rabbit UCP1 (Boster, CA). Secondary antibodies conjugated with horseradish peroxidase and chemiluminescence reagent (Amersham, Marlborough, MA) were used to visualize the target proteins. Densities of target protein bands were determined with Quantity One 4.4.1 (Bio-Rad, Hercules, CA). The internal control, β-actin, was used to normalize loading variations.

To assess the leptin protein expression in adipose tissue, lysates were prepared from epididymal adipose tissues using RIPA buffer (Sigma, St. Louis, MO) supplemented with protease and phosphatase inhibitors (Sigma, St. Louis, MO), and their leptin protein levels were assessed with ELISA kit (RayBio Mouse Leptin ELISA Kit, RayBiotech) per manufacturer’s instruction. The results were normalized by the concentration of total proteins, and presented as the percentage of the level in VV group.

### Statistics

All data are expressed as means ± SEMs unless noted otherwise. Statistical tests were performed using one-way or two-way analysis of variance (ANOVA) followed by Bonferroni correction or unpaired *t*-test using GraphPad Prism (version 5; GraphPad Software, La Jolla, CA, USA). The significance level was set at *p* < 0.05.

## Results

### Differential developmental programming by prenatal and postnatal mothering of DEP-exposed dams

**Fig. 1:**
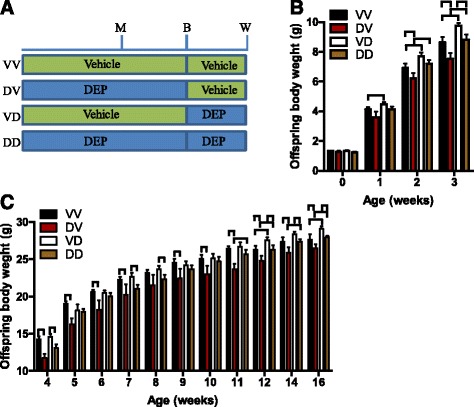
Differential developmental programming by prenatal and postnatal mothering of DEP-exposed dams. **a** Experimental scheme: M, initiation of mating; B, birth; W, weaning. **b** The growth trajectory of offspring during the lactation period. *n* = 28–39/group. ^⊓^ *p* < 0.05, ANOVA. **c** The growth trajectory of male offspring after weaning. *n* = 12–18/group. ^⊓^ *p* < 0.05, ANOVA

To document the long-term effects of maternal exposure to DEP on offspring development, dams (female C57/Bl6j mice) were treated with DEP or vehicle from the age of 5 weeks until weaning of offspring. Table 2 shows that this DEP exposure did not significantly alter the body weight of dams. As both prenatal and postnatal periods have been shown to be vulnerable to developmental programming [2], and one of the main objectives of the present study is to determine the window of developmental programming by maternal exposure to DEP, half offspring were switched between vehicle- and DEP-exposed dams once born. Thus, there were four groups of offspring in total (Fig. 1a): VV, offspring of vehicle-treated dams postnatally mothered by vehicle-treated dams; DV, offspring of DEP-treated dams postnatally mothered by vehicle-treated dams; VD, offspring of vehicle-treated dams postnatally mothered by DEP-treated dams; DD, offspring of DEP-treated dams postnatally mothered by DEP-treated dams. The comparisons of VV with DV and VD with DD reflected the effects of prenatal mothering by DEP-exposed dams, whereas the comparisons of VV with VD and DV with DD revealed the effects of postnatal mothering by DEP-exposed dams.

**Table 2:** The characterization of breeding results

	Dam weight (g)^a^	Pregnancy duration (days)^b^	Litter size	Sex ratio (M/F)
Vehicle	25.02 ± 0.52	25.8 ± 3.0	6.2 ± 0.5	1.5
DEP	24.92 ± 0.54	27.8 ± 2.1	6.75 ± 2.5	1.47

^a^dam weight was measured when euthanized; ^b^duration from the initiation of mating to the birth of offspring

**Fig. 2:**
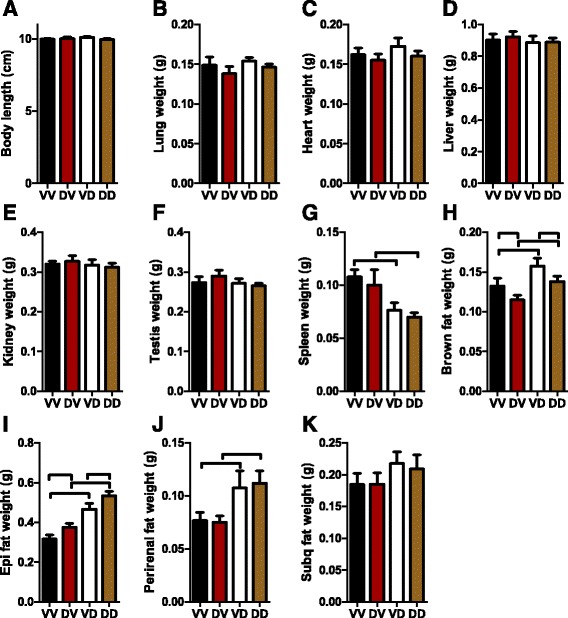
Prenatal and postnatal mothering by DEP-exposed dams differentially impact organ weights. Mice were euthanized at the age of 24 weeks. Their body length (**a**) and the weight of the indicated organs (**b**-**k**) were measured. ^⊓^ *p* < 0.05, ANOVA

Figure 1b shows that maternal exposure to DEP did not have a significant effect on offspring birth weight, suggesting that it does not impact the gestational durations. There may be critical periods during organogenesis particularly vulnerable to developmental programming. No difference of birth time relative to instillation time (born on the day of instillation: 4 litters in saline group versus 3 litters in DEP group, *n* = 6/group, *p* = 0.4, Fisher exact test.) strongly supported that their instillation timings relative to organogenesis were the same. Because we ever observed that maternal exposure to concentrated ambient PM_2.5_ altered body weights of adult male but not female offspring (data not shown), we did not follow up the growth of female offspring in the present study. Offspring hereafter means the male offspring only. Figure 1b shows that a significant weight gain effect of postnatal mothering by DEP-exposed dams (VV versus VD) was observed as early as postnatal week one. This effect maintained throughout the rest of lactation period (Fig. 1b), but vanished quickly after weaning. Notably, this weight gain effect of postnatal mothering by DEP-exposed dams re-appeared at postnatal week 11 and was maintained throughout the rest observation period, representing a typical developmental programming by environmental stressors. Figure 1b shows that prenatal mothering by DEP-exposed dams also had a significant effect on offspring body weight, but it rendered a weight loss after a latency of about two weeks. Unlike the weight gain effect of postnatal mothering by DEP-exposed dams, the weight loss effect of prenatal mothering by DEP-exposed dams was continuously observed since it emerged at postnatal week two (Fig. 1c). In contrast to their marked body weight effects, neither prenatal nor postnatal mothering by DEP-exposed dams significantly altered the offspring body length (Fig. 2a).

### Organ-specific effects of prenatal and postnatal mothering by DEP-exposed dams

To further document the developmental effects of prenatal and postnatal mothering by DEP-exposed dams, we measured the weights of main organs/tissues of offspring at the age of 22 weeks. Figure 2b-f show that neither prenatal nor postnatal mothering by DEP-exposed dams influenced the weights of lung, heart, liver, kidney, and testis in adult offspring. Figure 2g reveals that postnatal mothering by DEP-exposed dams significantly reduced the weight of spleen in adult offspring. Whereas it significantly increased the mass of brown adipose tissue (BAT, Fig. 2h), epididymal adipose tissue (Fig. 2i), and peri-renal adipose tissue (Fig. 2j). It also resulted in a trend of increase in the mass of subcutaneous adipose tissue (Fig. 2k). In contrast, prenatal mothering by DEP-exposed dams significantly decreased the mass of BAT, significantly increased the mass of epididymal adipose tissue, and did not significantly alter the weight of any other tested organ/tissue.

### Adipose effects of prenatal and postnatal mothering by DEP-exposed dams

**Fig. 3:**
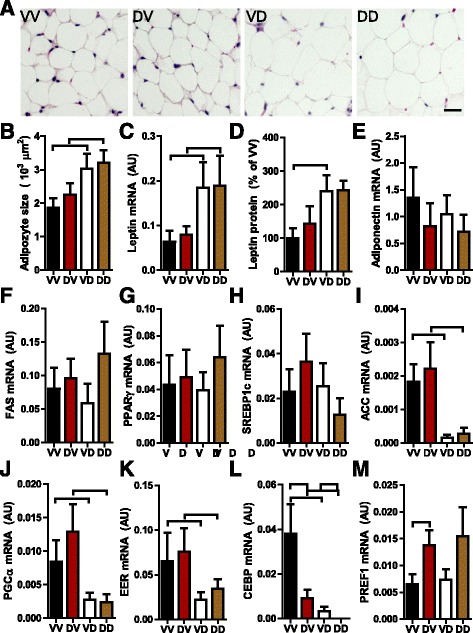
Prenatal and postnatal mothering by DEP-exposed dams differentially impact offspring adipose tissue. **a** and **b**, Epididymal adipose tissue was fixed, sectioned, and subjected to H&E staining. The representative images (**a**) and the quantitation data (**b**) are presented. ^⊓^ *p* < 0.05, ANOVA. **c**, the mRNA expression levels of leptin in epididymal adipose tissue were assessed by real-time RT-PCR. **d**, the protein levels of leptin in epididymal adipose tissue were assessed by ELISA. **e**-**m**, the mRNA expression levels of the indicated gene in epididymal adipose tissue were assessed by real-time RT-PCR. ^⊓^ *p* < 0.05, ANOVA

Obesity is one of the leading global health concerns. Given the marked effects of prenatal and postnatal mothering by DEP-exposed dams on adiposity of adult offspring, we performed histological assessments of epididymal adipose tissues. Figure 3a and b demonstrate that although both increased the mass of epididymal adipose tissue, postnatal but not prenatal mothering by DEP-exposed dams significantly increased the average size of adipocytes, suggesting that prenatal and postnatal mothering by DEP-exposed dams have different mechanisms for their obesogenic effects. Leptin is one of the critical adipokines of which expression correlates to adipocyte size. Consistent with the morphological analysis, Fig. 3c reveals that postnatal but not prenatal mothering by DEP-exposed dams significantly increased the expression of leptin in epididymal adipose tissue, whereas neither prenatal nor postnatal mothering by DEP-exposed dams altered the expression of FAS and adiponectin (Fig. 3e and f), two other adipocyte markers. ACC, PGC1, C/EBP, and ERRα have been shown to promote adipogenesis. Figure 3g-l however demonstrate that their expression in epididymal adipose tissue was significantly reduced by postnatal but not prenatal mothering of DEP-exposed dams. PREF1 has been shown to be an inhibitor of adipogenesis. Figure 3m reveals that prenatal but not postnatal mothering by DEP-exposed dams increased the expression of PREF1 in epididymal adipose tissue. Together, these data revealed that prenatal and postnatal mothering by DEP-exposed dams differentially impact the expression profile of adipogenesis markers.

### Prenatal but not postnatal mothering by DEP-exposed dams reduces offspring food intake

**Fig. 4:**
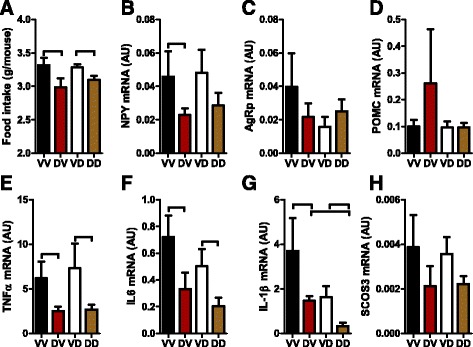
Prenatal and postnatal mothering by DEP-exposed dams differentially impact offspring food intake. **a**, average food intake of offspring for five consecutive days. ^⊓^ *p* < 0.05, ANOVA. **b**-**h**, the mRNA expression levels of the indicated gene in the hypothalamus were assessed by real-time RT-PCR. ^⊓^ *p* < 0.05, ANOVA

Alteration in body weight is an index of altered energy balance, which is determined by both intake and expenditure. Consistent with their body weight effects, Fig. 4a shows that prenatal but not postnatal mothering by DEP-exposed dams significantly reduced the food intake of offspring. Hypothalamus is the control center of food intake and regulates it primarily through the neural expression of orexigenic peptides such as Agouti-related peptide (AgRp) and Neuropeptide Y (NPY) and anorexigenic peptides such as pro-opiomelanocortin (POMC). Figure 4b shows that consistent with food intake effects, prenatal but not postnatal mothering by DEP-exposed dams significantly reduced hypothalamic expression of the orexigenic peptide, NPY. No any significant difference in hypothalamic expression of POMC and AgRp was observed (Fig. 4c and d). Hypothalamic inflammation has been shown to impact food intake through not yet identified mechanisms. Therefore, we also assessed the expression of pro-inflammatory cytokines in the hypothalamus. Figure 4e-h reveal that prenatal mothering by DEP-exposed dams significantly decreased the expression of TNFα, IL-6, and IL-1β in hypothalamus. In contrast, postnatal mothering by DEP-exposed dams had much smaller effects on the expression of those pro-inflammatory cytokines. It significantly decreased the expression of IL-1β only (Fig. 4g).

### Postnatal but not prenatal mothering by DEP-exposed dams increases offspring BAT whitening

**Fig. 5:**
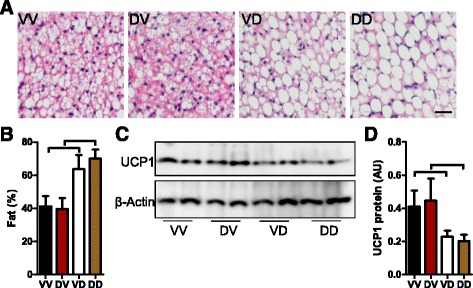
Prenatal and postnatal mothering by DEP-exposed dams differentially impact offspring BAT whitening. **a** and **b**, morphological analysis of mouse BAT. The representative images (**a**) and the quantitation of fat droplet area (**b**) are presented. *p* < 0.05, ANOVA. C and D, UCP1 protein levels in BAT were determined by western blot, and the representative images (**c**) and the quantitation of protein levels (**d**) are presented. ^⊓^ *p* < 0.05, ANOVA

BAT is the key thermogenic tissue that regulates energy expenditure. As shown in Fig. 2g, the prenatal mothering by DEP-exposed dams significantly decreased BAT mass, whereas the postnatal mothering by DEP-exposed dams increased BAT mass. Studies have shown that in addition to its mass, the “whitening” level of BAT, characterized by the accumulation of large lipid droplets and mitochondrial dysfunction, is a reflective of reduced energy expenditure. Figure 5a and b demonstrate that postnatal mothering by DEP-exposed dams markedly increased the size of lipid droplets (Fig. 5a) and also the total accumulation of lipid droplets in BAT (Fig. 5b). Consistent with morphological alterations, postnatal mothering by DEP-exposed dams significantly decreased the protein level of UCP1, the primary mitochondrial uncoupling protein of BAT. In contrast, prenatal mothering by DEP-exposed dams altered neither the accumulation of lipid droplets nor the expression of UCP1 in BAT.

## Discussion

The main findings in the present study include that 1) both prenatal and postnatal mothering by DEP-exposed dams programs offspring energy metabolism; 2) the programing of energy metabolism by them however are markedly different: while prenatal mothering by DEP-exposed dams leads to a weight loss, postnatal mothering by DEP-exposed dams results in a weight gain; 3) prenatal mothering by DEP-exposed dams specifically increased the mass of epididymal adipose tissue through hyperplasia, whereas postnatal mothering by DEP-exposed dams increased the mass of all tested fat pads through hypertrophy; 4) while prenatal mothering by DEP-exposed dams programs offspring energy balance primarily through reduction in food intake, postnatal mothering by DEP-exposed dams influence offspring energy balance primarily through induction of BAT whitening.

Abnormal energy metabolism is central in the pathogenesis of numerous diseases such as obesity, diabetes, and cancer. Exposure to ambient PM_2.5_ pollution has been shown to cause a variety of abnormalities in energy metabolism that may contribute to the pathogenesis of cardiometabolic diseases [26]. The present study further reveals that maternal exposure to DEP causes marked long-term effects on offspring energy metabolism through an exposure timing-dependent manner. As DEP are an important constituent of ambient PM_2.5_ in many urban areas such as New York City [27], those data thus markedly extend our understanding of ambient pollution-induced energy metabolic effects, but also provoke transgenerational health concerns over maternal exposure to ambient pollution.

In the present study, we demonstrate that DEP exposure covering a 7-week pre-conception period and the whole gestation did not significantly change the birth weight of offspring but led to a weight loss observed as early as two weeks after birth. This perfectly replicates the previous inhalation exposure study using DEP from the same source [28], validating our exposure method in assessing DEP toxicity. These data however are inconsistent with another inhalation exposure study showing that gestational DEP exposure reduces offspring birth weight [13]. Notably, DEP used in the latter is from different source, suggesting that the composition of DEP may play a critical role in determination of its effects on offspring.

To our knowledge, the present study is the first one providing the birth-to-adult growth trajectory of offspring mothered by DEP-exposed dams. Extending previous knowledge [28], the growth trajectory shows that the body weight effects of maternal exposure to DEP are long-lasting. This is perfectly consistent with the DOHaD paradigm [2]. Latency between exposure and disease/dysfunction is another important component of the DOHaD paradigm. In agreement with the DOHaD paradigm, the present study shows an obvious latency between maternal exposure to DEP and a variety of metabolic abnormalities in offspring. As such, our data reaffirm that maternal exposure to DEP is an environmental stressor for developmental programming of cardiometabolic diseases, raising more serious concerns about its transgenerational effects.

The present data show that prenatal maternal exposure to DEP reduced offspring food intake but not BAT whitening, accompanied by decreased hypothalamic expression of an orexigenic neuropeptide NPY, suggesting that prenatal maternal exposure to DEP may primarily program energy intake. These data are consistent with numerous studies showing that maternal exposure to environmental stressors exerts long-term energy metabolic effects on offspring through programming of hypothalamic circuits regulating energy balance [21]. Further studies are undergoing to identify the structural and/or functional alterations in the hypothalamus that may account for the decreased expression of NPY and food intake.

It is noteworthy that the present study also demonstrates that prenatal maternal exposure to DEP significantly reduced the hypothalamic expression of several pro-inflammatory cytokines including TNFα, IL-6 and IL-1β. Rapidly increasing evidence has indicated that hypothalamic inflammation plays a critical role in regulation of energy balance. Current evidence suggests that the relationship between hypothalamic inflammation and energy balance is context-dependent: while hypothalamic inflammation observed in many severe chronic illnesses reduces food intake and leads to a negative energy balance, obesity-associated hypothalamic inflammation has been shown to increase food intake [29]. The mechanism whereby hypothalamic inflammation context-dependently regulates food intake has not yet fully understood. It has been believed that the different effects of hypothalamic inflammations on energy balance reflect their different levels of inflammation in hypothalamus: while high level of inflammation reduces food intake, low level of inflammation increases food intake. Therefore, the decrease of hypothalamic inflammation in offspring prenatally exposed to DEP may be responsible for their reduction in food intake and consequent lower body weight. Further studies are thus warranted to determine the role of this decreased hypothalamic inflammation in programming of energy balance by prenatal maternal exposure to DEP.

It is particularly notable that although prenatal maternal exposure to DEP decreased offspring body weight, it paradoxically increased the mass of epididymal adipose tissue. These data somehow are consistent with lineage tracing studies showing that the pool of murine white adipocyte precursors is largely committed prenatally or just after birth [30], and thus warrant additional studies to examine the nature of insult of adipocyte precursors caused by prenatal maternal exposure to DEP. Interestingly, the present study also demonstrates that the adipose effect of prenatal maternal exposure to DEP appeared to be epididymal adipose tissue-specific. This reminisces about the heterogeneity of developmental origins of adipocytes [31], providing another piece of evidence that prenatal maternal exposure to DEP may damage early process of adipogenesis.

As no hypertrophy was observed, this increased mass of epididymal adipose tissue appears to be primarily due to hyperplasia. Animal adipocyte numbers have been shown to increase through puberty but be relatively steady in the mature fat pad [32, 33]. By far, how adipocyte numbers in the mature fat pad are regulated is not yet fully understood. Thus, the demonstration of maternal DEP exposure-induced adipose hyperplasia in adult offspring provides a valuable animal model to investigate how adipocyte numbers in the mature fat pad are regulated.

Another important finding in the present study is the demonstration of different energy metabolic effects of prenatal and postnatal mothering by DEP-exposed dams. Notably, although it is not uncommon to find that different timings lead to different consequences in developmental programing studies, to our knowledge, this is the first toxicological study showing that the timing of exposure to PM_2.5_ completely determines its long-term effects. Contrary to the most effects of prenatal mothering, postnatal mothering by DEP-exposed dams increased offspring body weight, did not impact their food intake but induced their BAT whitening. Those data together suggest that postnatal mothering by DEP-exposed dams induces a positive energy balance primarily though reduction in energy expenditure. Our data somehow are consistent with previous studies showing that postnatal catch-up growth programs susceptibility to obesity and impairment of BAT function in both humans and animal models [21, 34, 35]. Although still controversial, increasing evidence supports that brown adipocytes in both BAT and WAT (also known as “beige” component) contributes to regulation of human energy homeostasis [36]. Therefore, our demonstration of BAT whitening programed by maternal exposure to CAP in mice may be relevant to regulation of energy homeostasis in humans.

Additionally, the present data reveal that the postnatal mothering by DEP-exposed dams increases the adiposity of offspring, which appears to be primarily consequent to hypertrophy. Notably, the adipose hypertrophy is accompanied by decreased expression of several adipogenesis markers. This is perfectly consistent with previous studies showing that high fat diet treatment induces adipose hypertrophy and meanwhile decreases the expression of adipogenesis markers [37]. These data together suggest that the decreased expression of adipogenesis markers may be the consequence but not the cause of hypertrophy. Therefore, additional studies are still needed to delineate the mechanisms for this induction of adipose hypertrophy by the postnatal mothering by DEP-exposed dams.

## Conclusion

In conclusion, the present study demonstrates the timing-dependency of developmental programming by mothering of DEP-exposed dams. These different long-term effects of prenatal and postnatal mothering by DEP-exposed dams underscore consideration of the exposure timing when examining the adverse effects of maternal exposure to ambient PM_2.5_.

## Abbreviations

AgRp: Agouti-related peptide
BAT: Brown adipose tissue
BMI: Body mass index
DEP: Diesel exhaust PM_2.5_
DOHaD: The developmental programming of health and diseases
IL-1β: Interleukin 1beta
IL-6: Interleukin 6
NPY: Neuropeptide Y
PM_2.5_: Particulate matter with an aerodynamic diameter ≤ 2.5 μm
POMC: Pro-opiomelanocortin
TNFα: Tumor necrosis factor alpha
UCP1: Uncoupling protein 1
